# Evaluating the efficacy of a landscape scale feral cat control program using camera traps and occupancy models

**DOI:** 10.1038/s41598-018-23495-z

**Published:** 2018-03-28

**Authors:** Sarah Comer, Peter Speldewinde, Cameron Tiller, Lucy Clausen, Jeff Pinder, Saul Cowen, Dave Algar

**Affiliations:** 1 Department of Biodiversity, Conservation and Attractions, South Coast Region, 120 Albany Hwy, Albany, Western Australia 6330 Australia; 2 University of Western Australia, Centre of Excellence in Natural Resource Management, 6330 Albany, Western Australia Australia; 3 Department of Biodiversity, Conservation and Attractions, Science and Conservation Division, Woodvale, 6026 Western Australia Australia

## Abstract

The impact of introduced predators is a major factor limiting survivorship and recruitment of many native Australian species. In particular, the feral cat and red fox have been implicated in range reductions and population declines of many conservation dependent species across Australia, including ground-nesting birds and small to medium-sized mammals. The impact of predation by feral cats since their introduction some 200 years ago has altered the structure of native fauna communities and led to the development of landscape-scale threat abatement via baiting programs with the feral cat bait, Eradicat. Demonstrating the effectiveness of broad-scale programs is essential for managers to fine tune delivery and timing of baiting. Efficacy of feral cat baiting at the Fortescue Marsh in the Pilbara, Western Australia was tested using camera traps and occupancy models. There was a significant decrease in probability of site occupancy in baited sites in each of the five years of this study, demonstrating both the effectiveness of aerial baiting for landscape-scale removal of feral cats, and the validity of camera trap monitoring techniques for detecting changes in feral cat occupancy during a five-year baiting program.

## Introduction

Many species of Australian native mammals have experienced significant range declines or extinction in the arid zone since European settlement, and many species of birds have suffered similar declines over the same time period^1–7^. One of the often cited causes for this decline in native species is predation by introduced predators, in particular the feral cat (*Felis catus*) and red fox (*Vulpes vulpes*)^1,2,7^. In the Pilbara region of Western Australia, feral cats are widespread while foxes are largely confined to the coastal plain^8,9^.

Baiting is recognised as the most effective method for controlling feral cats^10–13^ in particular when there is minimal risk posed to non-target species. Effective and cost-efficient control of feral cats in large reserves, which often have restricted access, can only be achieved through aerial baiting campaigns^11,14^. The expansiveness of Pilbara landscapes necessitates landscape-scale abatement of introduced predators, as small-scale localised control programs are unlikely to achieve conservation objectives.

Monitoring the abundance of feral cats, like many mammalian carnivores, is difficult because they occur at low densities, have large home ranges and tend to be secretive and cryptic^15–19^. Capture-recapture studies to estimate abundance are usually impractical as cats are difficult to trap, leading to low capture rates and recapture probabilities^19^. Consequently, most monitoring schemes rely on indices of abundance.

Systematic monitoring of introduced predator activity before and after treatment is essential to demonstrate both effectiveness of baiting and to provide guidance to managers on optimal baiting strategies for the target biomes^20,21^. Feral cat control programs around Australia monitor baiting efficacy through various methods such as deaths attributed to poisoning of radio-collared animals, with supporting evidence often obtained through activity indices from counts of prints on sand-pads^11,22–26^. While the use of radio-telemetry to assess baiting efficacy is not limited by access restrictions, the use of sand-pads requires not only sufficient length of tracks of suitable substrate to reliably detect changes, but also unrestricted access to study areas^27^. Sand-pads also lend themselves to observer bias, and in areas where access can be problematic due to wet weather or restricted public access, they can be of limited value^27,28^.

Camera trap studies are useful in providing information on feral cat presence/absence but in many cases individuals cannot be easily distinguished with certainty^29,30^. At a rudimentary level, presence/absence data from camera trap sites can be used to provide indices of relative activity^31^. Raw detection rates (i.e. total number of sites where cats were detected/number of sites) are naïve estimates of occupancy that do not account for probability of detection^32^. If detection probabilities are determined, estimates of occupancy can be derived from presence/absence data^33,34^. Occupancy is often used as a metric for estimating various species’ occurrence and is a function of abundance as it concerns the probability of a particular animal being at a given site^32,33,35^. Rather than giving an estimate of population size, occupancy models calculate the probability of a site being occupied and provide a repeatable and objective means of evaluating the success of population knockdown in a structured decision-making framework^33,36,37^.

Camera trapping provides a method of supporting efficacy of bait uptake, and can provide a robust and repeatable measure that also improves detectability of introduced predators before and after baiting programs. Due to the difficulty of reliably identifying individual cats from photographs, consistent population estimates can be difficult to calculate. Occupancy modelling provides an alternative way of monitoring animal populations as they respond to management intervention, and is a method that has been tested in a limited number of short-term introduced predator control programs in Australia^38–41^. Therefore, in a baiting program, changes in site occupancy can be used to measure the effectiveness of baiting programs in an adaptive management framework.

This study aimed to determine the usefulness of camera traps for establishing feral cat baiting efficacy using Eradicat baits in the Fortescue Marsh, Western Australia^42^. Repeat camera survey data were used to establish if site occupancy models could be reliably used to demonstrate baiting effectiveness.

## Results

**Figure 1:**
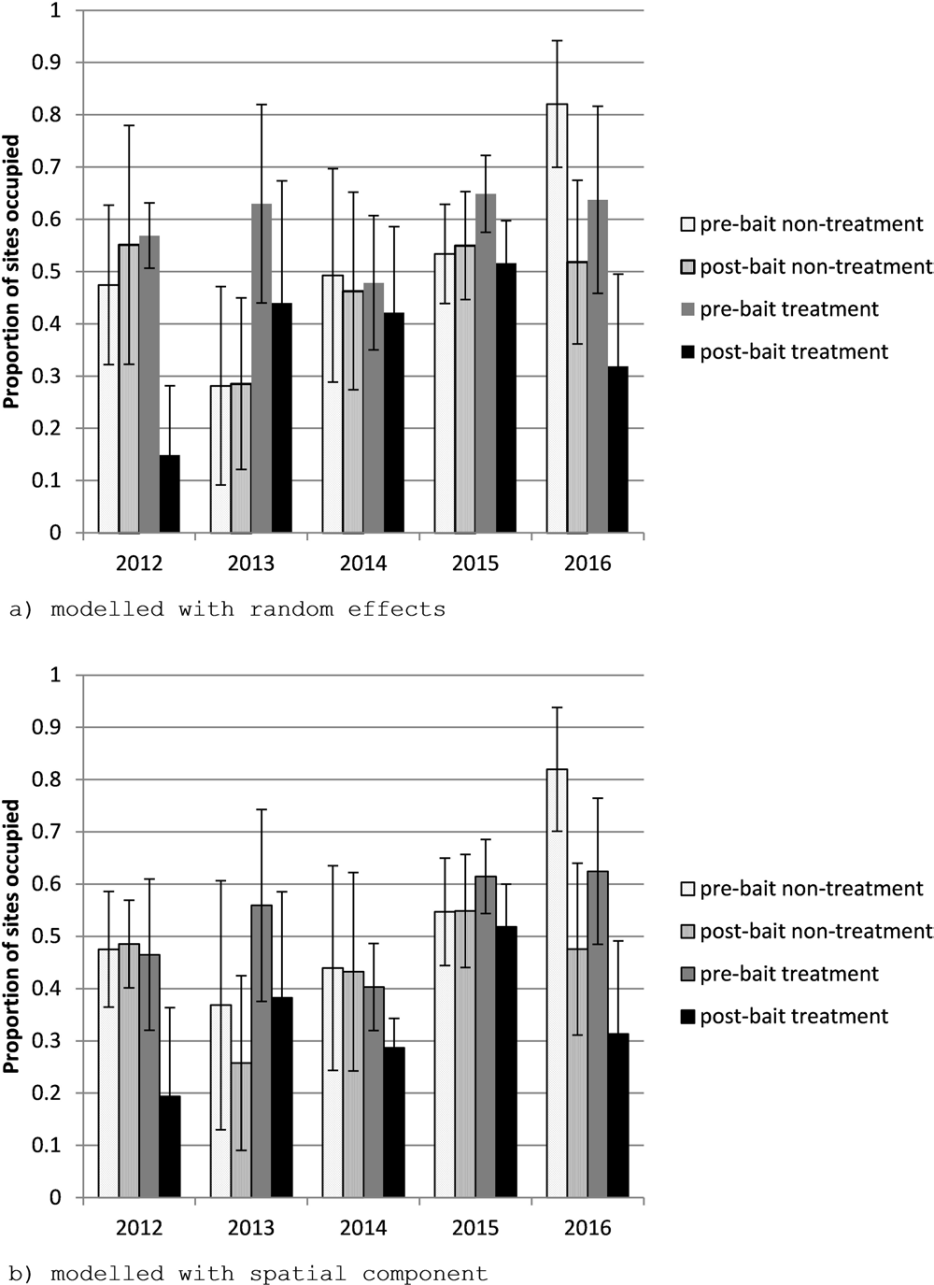
Modelled proportion of sites occupied (mean ± SD) in Fortescue Marsh treatment cell pre- and post-baiting for 2012, 2013, 2014, 2015 and 2016 with (**a**) random effects and (**b**) spatial component. Graphs were generated in Microsoft Excel 14.0.7180.5002 (https://products.office.com/en-au/excel).

For all years, cat activity at the treatment sites decreased post-baiting, measured as either number of cameras detecting cats (Table 1) or as occupancy, represented by the proportion of occupied sites across the study area (Fig. 1). The occupancy of non-treatment and treatment sites was calculated before and after baiting for data from all years (2012, 2013, 2014, 2015 and 2016) using both random effects (Table 2) and spatial models (Table 3). For all years there was a significant decrease in the calculated occupancy post-baiting in the treatment site using the random effects model (t-test, 2014 p = 0.043; other years p < 0.001) while occupancy in the non-treatment site did not change significantly in the four years between 2012 and 2015 (t-test, 2012 p = 0.17; 2013 p = 0.93, 2014 p = 0.56; 2015 p = 0.56) (Fig. 1). In 2016 the decrease in occupancy in the non-treatment cell following baiting was significant (t-test, p < 0.0001) although the extent of the decline was lower than that observed in the treatment cell.

**Table 1:** Number of cameras which detected cats (number of trap-nights). The non-treatment is an unbaited control, and treatment is the Eradicat bait cell.

	Pre-bait		Post-bait		Percentage change in camera trap success (relative to pre-bait)	
	Non-treatment	Treatment	Non-treatment	Treatment	Non-treatment	Treatment
2012	8 (791)	12 (1387)	4 (228)	2 (448)	73%	−48%
2013	3 (1103)	8 (630)	8 (1444)	4 (928)	104%	−66%
2014	6 (810)	16 (1482)	6 (750)	12 (1322)	8%	−16%
2015	5 (656)	13 (1152)	5 (552)	8 (993)	19%	−29%
2016	15 (780)	24 (1151)	8 (630)	7 (946)	−34%	−65%

**Table 2:** Results for random effects models for each year of the study, with t-values, degrees of freedom and p-values for both non-treatment (control) and baited treatment.

year	non-treatment					treatment				
	pre-bait	post-bait	t	df	p	pre-bait	post-bait	t	df	p
2012	0.4745	0.5511	1.4083	47	0.1656	0.5687	0.1491	19.8646	91	0.0001
2013	0.2813	0.2854	0.091	60	0.9278	0.6298	0.4399	3.4197	57	0.0012
2014	0.4927	0.4628	0.5884	58	0.5585	0.4785	0.4215	2.047	110	0.043
2015	0.534	0.5497	0.5812	52	0.5636	0.6487	0.516	8.0321	86	0.0001
2016	0.8206	0.5182	8.3676	58	0.0001	0.6374	0.3186	10.1484	126	0.0001

**Table 3:** Results for spatial models for each year of the study, with t-values, degrees of freedom and p-values for both non-treatment (control) and baited treatment.

Year	non-treatment					treatment				
	pre-bait	post-bait	t	df	p	pre-bait	post-bait	t	df	p
2012	0.4753	0.4852	0.3384	47	0.7366	0.4649	0.194	8.3073	91	0.0001
2013	0.3684	0.2577	2.1181	60	0.0383	0.5593	0.3824	3.5067	57	0.0009
2014	0.4393	0.4325	0.1366	58	0.8918	0.4031	0.287	8.6732	110	0.0001
2015	0.5472	0.5487	0.0521	52	0.9586	0.6147	0.5181	5.9147	86	0.0001
2016	0.8198	0.4755	9.3007	58	0.0001	0.6246	0.3134	11.011	126	0.0001

A similar result was obtained using a model incorporating spatial effects, where there was a significant decrease in the occupancy of the treatment site in all years (t-test p < 0.001). When modelled using the spatial effects model there was no significant change in occupancy for the non-treatment site for three years (t-test, 2012 p = 0.74; 2014 p = 0.89; 2015 p = 0.96). In 2013 and 2016 there was a significant decrease in occupancy for the non-treatment site (t-test, 2013 p = 0.04; 2016 p < 0.0001). The size of the decrease in the non-treatment site in 2013 (decrease of 0.1107) was less than in the treatment site (decrease of 0.1769). In 2016, the probability of occupancy in the non-treatment cell was unusually high pre-baiting (0.8198) and the decline in occupancy recorded in the non-treatment was slightly higher than the treatment (Table 3). The frequency of calculated of camera locations occupied was not significantly different from the frequency of sites where cats were detected (random effects model Chi^2^ = 7.4, p > 0.99, spatial model Chi^2^ = 10.6, p > 0.95).

Of the 65 feral cats radio collared during the project only 33 had datasets where home range estimates reached an asymptote over the deployment period and only these were used for further analysis. The mean home range of collared feral cats using the minimum convex polygon method (MCP95) was variable across years, with male home ranges varying from 148 ha to 15553 ha (Table 4). Female home range size was between 104 and 1974 ha (Table 4). Mean home range size decreased over the study, with the areas occupied in 2016 on average lower than previous years (Table 4).

**Table 4:** Summary of home range size, percentage of cameras in each territory and potential interception of radio-collared cats with cameras in 2014, 2015 and 2016.

Year	Sex	Home Range MCP95% (ha)	%camera sites within 95% MCP home range	#cameras cat passed with 50 m radius
2014	Male (5)	5093.28 ± 5305.78	7.86 ± 7.86	1.80 ± 1.17
2014	Female (4)	689.10 ± 285.63	1.60 ± 1.10	0.75 ± 0.38
2015	Male (6)	3099.57 ± 1487.6	3.78 ± 2.50	1.50 ± 1.12
2015	Female (5)	1304.64 ± 621.78	2.26 ± 2.01	0.60 ± 0.80
2016	Male (5)	1175.94 ± 1177.17	1.58 ± 2.44	0.80 ± 0.75
2016	Female (8)	374.70 ± 205.58	0.80 ± 1.67	0.63 ± 0.48

One of the assumptions of occupancy models is that an animal is not recorded at more than one site. Data from the 33 radio collared cats in 2014, 2015 and 2016 indicated that although the sites were located 3 km apart (a distance estimated to achieve spatial independence between cameras) between 30% (2014), 40% (2015) and 61% (2016) of the collared cats were likely to have passed close (within a 50 m radius) to more than one camera (Table 4). Over the three years where comprehensive collar data were collected (2014, 2015 and 2016) only one of the collared cats was photographed by a camera, despite the majority of collared cats being in the vicinity of the cameras on at least one occasion during the camera monitoring period.

In 2014, mortality of six of the seven radio-collared cats was attributed to bait consumption, and occurred within 23 days of baiting. The seventh collared cat in 2014 was euthanized, having survived the baiting. Mortality of four of eleven collared cats was attributed to baiting in 2015 and nine of eleven in 2016. The surviving cat from 2014, and six out of seven bait survivors from 2015, were euthanized one month post-baiting. Extrapolating the mortality figures to the larger population, 85% of feral cats in the baited area died as a result of baiting in 2014, 36% in 2015 and 82% in 2016.

## Discussion

The large home ranges, cryptic nature and general low densities of feral cats present significant challenges for managers trying to monitor effectiveness of control programs^17,19^. The use of cameras provides an alternative to trapping, track counts and direct bait uptake to measure changes in feral cat populations pre- and post-treatment. This study showed that infrared cameras can be utilised to monitor the efficacy of landscape-scale feral cat control. Over the five years of this study in the Fortescue Marsh ecosystem, the use of cameras provided sufficient data to detect significant change in site occupancy following a baiting program.

In most situations the use of a non-treatment site as a ‘control’ was able to validate the impact of baiting, in that there was no significant difference in the pre- and post-bait occupancy in the unbaited non-treatment cell. However, in 2016 the high pre-bait occupancy and apparent post-bait decline in the non-treatment cell was contrary to this. It is possible that the delay in delivering baits which followed pre-bait monitoring, and subsequent delay in post-bait monitoring may have confounded the 2016 results. This highlights some of the issues with violating the premise of a closed system, which is likely to be a problem at times when animals are breeding. The 2016 decline in the non-treatment may have been the result of female cats shifting to being more sedentary with young^43^, which highlights the problems with monitoring efficacy of baiting programs during breeding periods potentially confounding results. Despite this situation, there was still a substantial decline in occupancy in the treatment cell as for previous years, and the camera data were supported by mortality of radio-collared cats.

The results of this study indicate that baiting, while not 100% effective, can have a significant impact on feral cat activity. The potential benefits of an effective and broad-scale feral cat control program to the biodiversity values of the Fortescue Marsh area are significant; with the marsh containing habitat for a number of threatened mammals and ground-nesting and migratory birds. Landscape-scale baiting programs are the only currently available mechanism to implement control in areas of this size, but it is imperative that the success or failure of the program can be effectively gauged. Being able to measure the effectiveness of baiting programs is critical not only to improving and adjusting delivery mechanisms for optimal introduced predator control, but also to understanding the impact of baiting, and to justify the cost of broad-scale bait delivery^11,29^.

Our study also supports the utilisation of camera traps for monitoring the response of a feral cat population to a baiting program over multiple years. Over the three years (2014–2016) where feral cats were radio-tracked only one radio collared cat was detected by a camera, indicating that the cameras only detect a proportion of the population; which in this case provided sufficient data for robust occupancy modelling. It is possible that the human scent associated with camera deployment and servicing may act as a deterrent for previously captured cats that may be close enough to be detected on camera. Camera trap avoidance behaviour may also be associated with the trapping of individuals to attach collars. The use of dissociative anaesthetics^44,45^ when cats are to be released for collecting telemetry data might improve the probability of detecting these animals on camera if avoidance of cameras is associated with prior capture or human contact.

Improving the probability of detecting the target species is highly desirable. This becomes particularly relevant after sustained removal programs and an expected decline in the target animal following successful control programs. Extending the period of camera deployment could overcome this problem; however, the longer the monitoring period the greater the probability that the assumption of the system being ‘closed’ will be violated^35,46^. This could be overcome by increasing camera numbers across a larger treatment area, or improving the attractiveness of the lures to attract more animals in the system; however, the practicalities of resourcing and managing large numbers of cameras may make this impractical for conservation managers^46^.

Over the five years of this project, the proportion of sites occupied declined following baiting in the treatment cell. Both random effects and spatial occupancy models were utilised in this study to measure changes in feral cat activity. One of the assumptions of occupancy models is that individuals cannot be recorded at more than one location^33^. Camera sites were placed such that there was a low probability of individual cats appearing on more than one camera. The GPS-tracking data showed that although there were multiple cameras within most collared cats’ home ranges only one of the 65 cats collared between 2014 and 2016 was recorded by a camera in the year of capture, and during the period of tracking. This would indicate that although there was a possibility of uncollared cats appearing on multiple cameras, the probability of this occurring was reasonably low given the distribution of cameras and feral cat home range size in this region. The low detection of collared cats on cameras also indicates that cat detection by cameras is a low probability event, which may have implications for monitoring of feral cat populations at low densities during eradication programs. For this reason, the spatial model employed was considered a more reliable estimate of occupancy as this model accounts for any spatial autocorrelation between cameras.

While the proportion of sites occupied by feral cats decreased following baiting in all years, the proportion of sites occupied between years showed little variation. This is indicative of an open-system population of feral cats, which is one of the challenges in implementing control programs over large landscapes, in particular those with a large perimeter:area ratio like Fortescue Marsh. Studies at Lorna Glen, another arid ecosystem in Western Australia, found that indices of cat activity increased six months after baiting, and the majority of reinvasion was associated with natal recruitment and immigration of juveniles from outside of the baited area^14^. Genetic monitoring may provide an opportunity to further investigate the long-term impact of baiting on the dynamics of this population and work to this end is currently being undertaken (Cowen *et al*. unpublished data). For the Fortescue Marsh system future improvements to feral cat control programs might expand the treatment cell to encompass the Hamersley and Chichester Ranges, thereby decreasing the perimeter:area ratio and providing a buffer to the core conservation area. Other tools for controlling feral cats, such as hand baiting and trapping, could be considered to support the aerial baiting program.

In our study occupancy models using camera trap data have clearly demonstrated the efficacy of baiting programs over large areas in successive years. The use of these easily replicated techniques for measuring success or failure of such programs is of enormous value to conservation managers.

## Methods

### Site description

**Figure 2:**
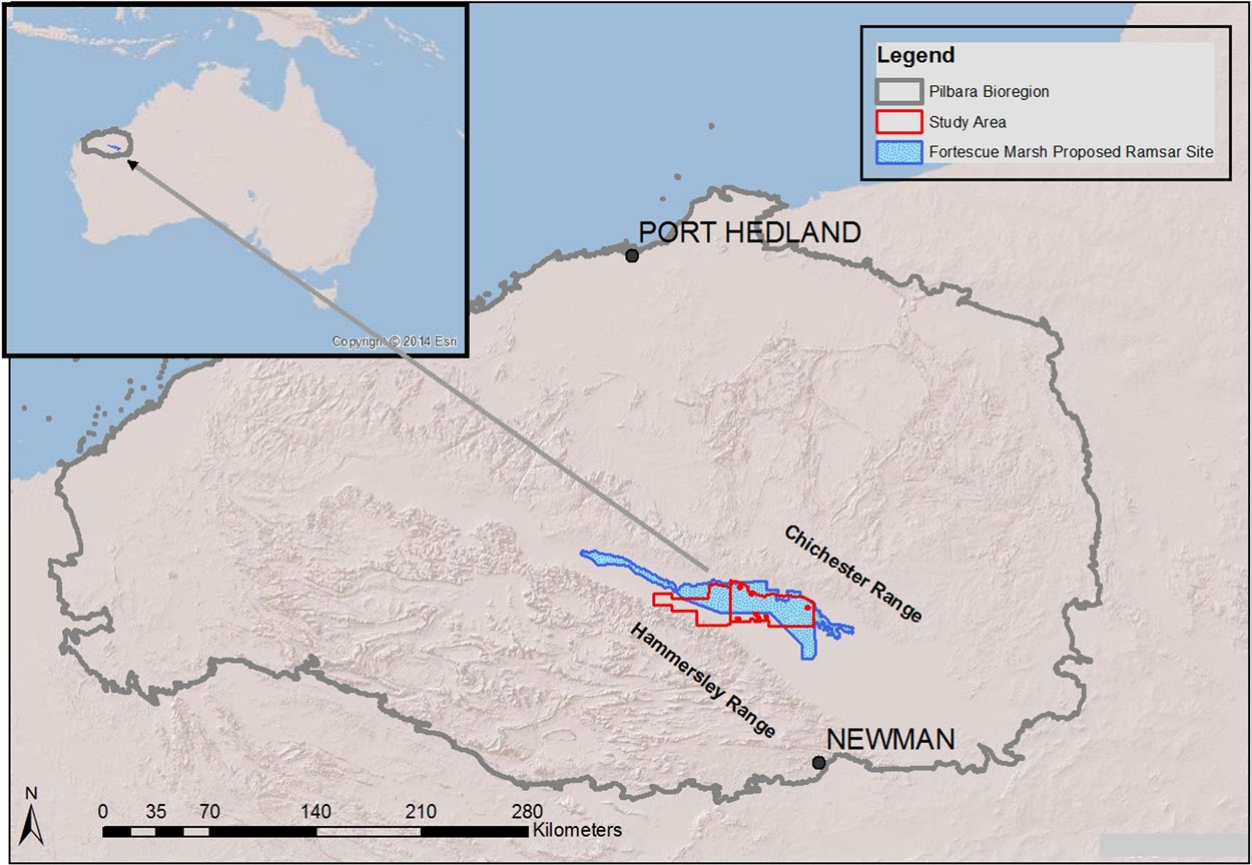
Location of Fortescue Marsh study site and Fortescue Marsh in the Pilbara bioregion, showing the wetland of international significance site. Map created using ArcMap 10.1 (www.esri.com).

Fortescue Marsh is the headwater of the Fortescue River and comprises a contiguous system of lakes, marshes and pools occupying the broad valley that lies in the Hamersley Basin of the Pilbara Craton. It is located in the Fortescue sub-region of the Pilbara biogeographic region, between the Chichester and Hamersley Ranges (Fig. 2)^8,47^. The marsh system extends approximately 200 km between these formations, and is dominated by scattered samphires and samphire heaths (*Tecticornia* spp.) and saltbush (*Atriplex* spp.) shrublands, shrubby grasslands dominated by salt water couch (*Sporobolus virginicus*) with false lignum (*Muellerolimon salicorniaceum*) and lignum (*Muehlenbeckia cunninghamii*) on floodplains. Fringing vegetation is dominated by low mulga woodlands and wattle shrublands^8^. The eastern half of the marsh, which is listed in the Directory of Important Wetlands in Australia, is up to 10 km wide, and the western half up to 3 km wide^48^. The basin fills intermittently, influenced by the tropical cyclone systems that predominantly occur in the Pilbara between January and March. When flooded, the marsh is considered to be highly significant for waterbirds^49^ and some sixty species, or 36% of the regional avifauna, depend partly or largely on the marsh^50^. A number of other conservation significant taxa, protected under the Australian *Environmental Protection and Biodiversity Conservation* (EBPC) Act (1999) have also been recorded from the area including the night parrot (*Pezoporus occidentalis*), brush-tailed mulgara (*Dasycercus blythi*) and bilby (*Macrotis lagotis*)^9,51^. Approximately 15% of the original mammal fauna of the region is extinct, due largely to the impact of introduced predators and changing land use^9^. Control of feral cats is necessary if the current suite of native fauna is to persist in these landscapes.

The study area lies within the eastern section of the marsh system, and comprises an area of approximately 1600 km^2^ (Fig. 2). The study site has been divided into a treatment cell of approximately 1020 km^2^, and an unbaited non-treatment (or control site) of 400 km^2^, with a 5 km wide buffer of around 180 km^2^ between the two^2,42^.

### Baits and baiting application

The Eradicat bait was developed specifically for the control of feral cats^11,12^. Eradicat baits contain 4.5 mg of directly injected toxin, sodium monofluoroacetate (compound 1080). Baits were deployed aerially from a dedicated baiting aircraft which released the baits at predetermined drop points to achieve desired application rate. The current recommended baiting density is 50 baits per km^2^ along flight transects 1 km apart to achieve a ground spread of approximately 200 × 40 m. Baiting was conducted in mid-winter when the weather conditions were cool and dry to maximise bait uptake. At this time, the abundance and activity of all prey types, in particular predator-vulnerable young mammals and reptiles, are at their lowest and bait degradation due to rainfall, ants and hot, dry weather is significantly reduced.

The Fortescue Marsh baiting program commenced in 2012 with a total area of 838 km^2^ baited with Eradicat. In 2013, a similar area was baited again, and in the three years between 2014 and 2016 a slightly larger area of approximately 920 km² was baited^42^. Changes to the actual baiting area were necessary due to water lying in the marsh and accessibility of sites. A BACI (Before-After Control-Impact) design was used to determine the impact of baiting on feral cats. The non-treatment area remained unbaited while the treatment area was baited. Both areas were monitored with camera traps prior to commencement of baiting and again post-baiting.

### Camera trapping

Reconyx Hyperfire HC600 (Reconyx, Wisconsin; USA) passive infrared camera traps were installed at a minimum of 44 locations in the baited area and 24 locations in the non-treatment to survey for the presence of feral cats (these numbers were variable due to access restrictions in some years). Camera sites were a minimum of 3 km apart to minimise the probability of individual cats appearing on more than one camera, in a grid array extending approximately 48 km by 16 km for the treatment and 11 km by 15 km for the non-treatment over the five years. Cameras were set to record five pictures per trigger with a picture interval of two frames per second. At each plot the camera was mounted 30 cm above the ground on a 45 cm heavy duty plastic peg. Cameras were all situated with a southerly aspect, and a 3 × 2 m pruned strip of vegetation with a lure station at the southern end of the clearing. Vegetation between camera and lure station was pruned to ground level to provide an uninterrupted view and minimise false triggers.

Cameras that do not have lures often generate sample sizes that are too low to adequately monitor population changes^29^ and the use of lures may also provide more precise population estimates by increasing the number of recaptures^52,53^. Therefore, a combination of olfactory and visual lures was used to attract feral cats to the camera traps. Lures for the camera trap surveys consisted of a small jar, with perforated lid, containing an oil-based scented lure (Catastrophic, Outfoxed Pest Control, Victoria) which was attached to a wooden stake approximately 30 cm from the ground. A 1.5 m long bamboo cane was joined to the wooden stake, with white turkey feathers connected to the cane approximately 30 cm above the scented lure. A 30 cm length of tinsel was fixed to the top of the stake in a position where it was not within the field of view of the camera.

Camera trap plots were established in 2012, and although the non-treatment site was shifted in 2013, the same sites were used for 2014, 2015 and 2016. Variation in water levels in the marsh resulted in some sites being relocated within the treatment cell; however, most sites were initially established at least two weeks prior to camera operation. Lures, memory cards and batteries were removed at the end of each survey period (i.e. before or after baiting) and reinstated and refreshed at the commencement of each survey period. At the time of installation, all cameras were test-fired to confirm functionality and correctness of aim. Cameras were operated for a period of up to 21 days before and after (or pre-and post-) baiting. Cameras and lures were removed a week prior to baiting and re-established two to three weeks after baiting. A ‘trap-night’ was the 24 hour period from mid-day (12:00 h) on day one to mid-day (12:00 h) the following day.

### Occupancy modelling

Occupancy models^33^ were used to determine the impact of baiting on feral cats. Detection of a species at a site confirms that the species is present at the site, but non-detection at the site does not necessarily mean that the species is absent^33^. Occupancy models use detection histories at sites to generate a probability of occupancy rather than just presence/absence. These probabilities are based on four assumptions^33^: population closure, no un-modelled heterogeneity in occupancy, no un-modelled heterogeneity in detection, and detection histories at each site are independent. To minimise the possibility of natural changes in the cat population the survey period was limited to approximately three weeks sampling immediately before baiting and approximately three weeks sampling commencing 21 days after the baiting period.

Occupancy models were used to examine the impact of baiting on the feral cat populations. Occupancy was calculated based on a basic occupancy model with the assumption of constant occupancy probability and constant detection probability during the period of camera trapping. Two forms of the model were used, to account for heterogeneity across the site a random effects component was included in one model. To allow for the possibility of a cat appearing on more than one camera a spatial component was included which accounted for the potential detection of cats at adjacent camera locations. Models were run using WinBUGS (v14)^54^. Winbugs code for both models are provided as supplementary material (S1). Both of the models (random effects and spatial) were run for the before control data, before impact data, after control data and after control data for each year to generate estimates of occupancy i.e. each year each site had an occupancy value estimated using the random effects model and an occupancy value estimated using the spatial effects model.

The models were run with a burn in of 5000 iterations before sampling for 5000 iterations for data collected both before and after baiting. The model calculated the proportion of occupied camera locations at each iteration, which was based on an average of all camera location occupancies. As this overall proportion of sites occupied was normally distributed the mean of this estimate from 5000 iterations was used as the value for proportion of occupied sites. This calculated value of proportion of occupied sites for the treatment and control areas was compared pre and post baiting using t-tests. Chi^2^ test was used to determine if the estimates for occupancy reflected the frequency of sites where cats were detected.

### Feral cat telemetry

In 2014, 2015 and 2016 feral cats over 1800 g were fitted with GPS-radio collars (Advanced Telemetry Systems, Minnesota, USA; Sirtrack, Havelock North, New Zealand). Based on the recommended ratio of collar size smaller cats (less than 1800 g) were considered too light for collaring, and released on site. Trapped cats were sedated with an intramuscular injection of 4 mg/kg Zoletil 100 (Virbac, Milperra, Australia). All animals captured were sexed, weighed, had coat colour recorded and were released at the site of capture. Collars were programmed to take 24 fixes per day (i.e. hourly intervals) during the baiting period, and deployed at least six weeks prior to baiting and prior to establishment of cameras. Individual collars were marked with a unique identifying symbol made from reflective-fluorescent tape to enable confirmation of individual identification if they were detected on a camera trap^55^. The home range of feral cats varies significantly across Australia, and may be influenced by season, sex, population density, prey availability and the type of habitat occupied^29,30,56–59^. Given the lack of knowledge of home range size in the Pilbara, data from collars which collected more than 500 data points (20 days) and where the home range estimates reached an asymptote over the deployment period, were used to estimate home range size and also establish the potential for animals to appear on more than one camera. Home range size was estimated using the 95% Minimum Convex Polygon model in QGIS (version 2.8.1) for fixes taken at hourly intervals, using the Animove plugin (Animove Team, AniMove – Animal Movement methods http://www.faunalia.it/animov/ (2008)).

### Ethics statement

This work was carried out under the Department of Parks and Wildlife Animal Ethics Permits 2012/42,2013/07,2015/39 and 2016/25 and all field methods followed procedures approved by the Western Australian Department of Biodiversity, Conservation Attraction’s Animal Ethics Committee.

## Electronic supplementary material

Supplementary Information
