# Supplementary material for: Evaluating the efficacy of a landscape scale feral cat control program using camera traps and occupancy models
Source: Sci Rep. 2018 Mar 28;8:5335. doi: 10.1038/s41598-018-23495-z (PMC5871771; doi:10.1038/s41598-018-23495-z)
Supplement: Supplementary file 1 — Supplementary Information [file 41598_2018_23495_MOESM1_ESM.doc]

Evaluating the efficacy of a landscape scale feral cat control program using camera traps and occupancy models: Authors: Sarah Comer, Peter Speldewinde, Cameron Tiller, Lucy Clausen, Jeff Pinder, Saul Cowen and Dave Algar

**Supplementary Information S1(methods)**: Winbugs code for the two different models used for analysis of remote camera data in Winbugs (v14): A) to allow for the possibility of a cat appearing on more than one camera a spatial component was included which accounted for the detection of cats at adjacent camera locations, and B) to account for heterogeneity across the site a random effects component was included in one model.

A) Spatial effects model

model {

for (i in 1:sites)

{

logit(psi[i] )<- PSI +sp[i]

z[i] ~ dbern(psi[i])

z1[i] <- z[i]+1

for (j in 1:K)

{

p[i,j,1] <- 0

logit(p1[i,j,2]) <- b[1] + re[i]

p[i,j,2]<-max(0.000001,min(0.99999,p1[i,j,2]))

X[i,j] ~ dbern(p[i,j,z1[i]])

}

}

psi.fs<-sum(z[])/sites

# CAR prior distribution for spatial random effects:

sp[1:sites] ~ car.normal(adj[], weights[], num[], tau.sp)

for(w in 1 : sumNumNeigh) {

weights[w] <- 1

}

## define prior distributions for model parameters

PSI ~ dnorm(0.0,0.37)

b[1] ~ dnorm(0.0,0.37)

## Priors for random effects

for(y in 1:sites) { re[y] ~ dnorm(0.0,det_tau) }

##detection

det_sigsq~dunif(0,6)

# variance component

tau.sp ~ dgamma(0.5, 0.0005)

det_tau<-pow(det_sigsq,-2)

}

B) Random effects (heterogeneous) Model

model {

for (i in 1:sites)

{

psi[i] <- PSI

z[i] ~ dbern(psi[i])

z1[i] <- z[i]+1

for (j in 1:K)

{

p[i,j,1] <- 0

logit(p[i,j,2]) <- b[1] + re[i]

X[i,j] ~ dbern(p[i,j,z1[i]])

}

}

psi.fs<-sum(z[])/sites

## define prior distributions for model parameters

PSI ~ dunif(0,1)

b[1] ~ dnorm(0.0,0.37)

## Priors for random effects

for(y in 1:sites) { re[y] ~ dnorm(0.0,det_tau) }

## detection

det_sigsq ~ dunif(0,6)

# variance component

det_tau <- pow( det_sigsq,-2)

}
